# The Intervention of Diet on Energy Metabolism

**DOI:** 10.3390/nu15112544

**Published:** 2023-05-30

**Authors:** Marina C. Oliveira, Adaliene V. M. Ferreira

**Affiliations:** Immunometabolism, Department of Nutrition, Nursing School, Universidade Federal de Minas Gerais, Belo Horizonte 30130-100, Brazil; marina.cdoliveira@gmail.com

Food intake patterns determine changes in energy expenditure due to their influence on body size and composition (percentage of fat, bone, and muscle), which can modulate signaling pathways that optimize energy consumption. On the other hand, energy restriction may cause an adaptive reduction in energy expenditure by lowering tissue metabolism and reducing body movement. This research topic aimed to highlight studies on the association between the effect of diet profile, nutrient supplementation, and/or eating habits on energy metabolism and, mainly, energy expenditure in metabolic disorders of any type. It conveys a collection of three original research articles, including articles particularly interested in the role of specific nutrients or supplements in pathogenic and physiological processes linked to energy metabolism.

García-Luna et al. [1] demonstrated the effect of intermittent fasting in rats subjected to a pair- or single-house stress condition treated with a palatable diet rich in calories for five weeks. Under stress conditions, rats fed a palatable diet increased energy intake and adipocyte size, which were associated with lower energy expenditure, as demonstrated by the reduced PGC1α and UCP1 expression. However, intermittent fasting reversed these parameters, favoring an increased energy expenditure and reduced body weight. They suggest that intermittent fasting controls the metabolic rate, supporting this regimen as a suitable non-pharmacologic strategy to treat obesity, even in stressed individuals. It modulates limbic dopaminergic and thyrotropin-releasing hormone systems that regulate feeding and hypothalamic–pituitary–thyroid axis function.

Another study, by Barzanjeh et al. [2], demonstrated the effects of alpha-glycerylphosphorylcholine (A-GPC), a food supplement, on heart rate variability (HRV) and hemodynamic responses following a sprint interval exercise in women with overweight or obesity. They hypothesized that the increased levels of free choline and acetylcholine that would result from consuming an alpha-glycerylphosphorylcholine supplement could play a protective role on cardiac autonomic and blood pressure in people with obesity when they are exposed to a bout of sprint interval exercise. Alpha-glycerylphosphorylcholine consumption recovers HRV and blood pressure faster following strenuous exercise in women with overweight and obesity. It was suggested that alpha-glycerylphosphorylcholine intake might favorably modify the cardiac autonomic function.

Lastly, a study by Gao et al. [3] described the effect of developmental diets (a low-yeast diet) on regulating the lifespan of adult Drosophila during development and adulthood. It was demonstrated that a developmental diet achieved the nutritional programming of the lifespan of adult males by modulating the activity of dFOXO in Drosophila. This molecule is required for lifespan extension and improved glucose metabolism phenotype. However, the knockdown of dFOXO in the fat abolished the lifespan-extending effects of a low-yeast diet during development, suggesting that fat may be an essential target for developmental nutritional programming. They proposed that the nutrition in the early life of animals could program their later life’s health and longevity by FOXO’s involvement.

The manuscripts presented in this research topic focused on different aspects of energy expenditure in the context of obesity and regulation of the lifespan by nutrients, considering animal models and human beings. Overall, this research topic provides a snapshot of this exciting field, highlighting the importance of nutritional components and dietary interventions in obesity modulation or its impact on longevity. Indeed, dietary interventions may be recognized as a potent tool to treat obesity and promote a healthy life.
